# A Systematic Review and Meta-Analysis on the Accuracy of Fluorodeoxyglucose Positron Emission Tomography/ Computerized Tomography for Diagnosing Periprosthetic Joint Infections

**DOI:** 10.3389/fsurg.2022.698781

**Published:** 2022-06-01

**Authors:** Mei Hu, Guangwen Chen, Lin Luo, Lan Shang

**Affiliations:** Department of Radiology, Sichuan Provincial People’s Hospital, Sichuan Academy of Medical Sciences, Chengdu, China

**Keywords:** meta-analysis, periprosthetic joint infection, positron emission tomography, validation studies, systematic review

## Abstract

**Objective:**

Fluorodeoxyglucose Positron emission tomography/computerized tomography (FDG PET/CT) has become popular for diagnosing periprosthetic joint infections (PJI). However, the diagnostic accuracy for this technique has varied from report to report. This meta-analysis was performed to assess the accuracy of FDG PET/CT for PJI diagnosis.

**Material and Methods:**

We conducted a systematic search of online academic databases for all studies reporting the diagnostic accuracy of FDG PET/CT for PJI. Meta-analysis was performed using STATA software.

**Results:**

23 studies, containing data on 1,437 patients, met inclusion criteria. Pooled sensitivity and specificity of FDG PET/CT for diagnosing PJI were 85% (95% CI, 76%, 91%) and 86% (95% CI, 78%, 91%), respectively with an AUC of 0.92. LRP was 6.1 (95% CI, 3.8, 9.7) and LRN was 0.17 (0.11, 0.28), indicating that FDG PET/CT cannot be used for confirmation or exclusion of PJI. There was significant inter-study heterogeneity, but no significant publication bias was noted.

**Conclusions:**

Our study found that FDG PET/CT has an important role as a diagnostic tool for PJI with high sensitivity and specificity. Further studies exploring its accuracy in different PJI locations remain necessary.

## Introduction

Alongside increasing life expectancies, the worldwide prevalence of adults aged 50 years or more living with a prosthesis has been estimated at 2.3%, with the proportion rising to 6% for individuals 80 years of age or older (1). In particular, joint arthroplasty incidence has increased substantially over recent decades (2). This has created an issue where a considerable portion of these prostheses must be revised within five or ten years (3). Common reasons for prosthetic revision include aseptic (55%) and septic loosening (7%), dislocation (12%), and periprosthetic fracture (6%) (4). While dislocations and periprosthetic fractures can be readily diagnosed, it is often challenging to differentiate aseptic from septic loosening (5). In the United States, two-stage exchange is the procedure of choice, while in European countries, one-stage procedure is preferred whenever feasible if the pathogen is known and the skin & bone are in good condition (6). Hence, this differentiation is very important clinically since the treatment of aseptic loosening follows either of these procedures and use of beads carries a risk of colonization (6).

American Academy of Orthopaedic Surgeons (AAOS) guidelines for periprosthetic joint infection (PJI) diagnosis recommends erythrocyte sedimentation rate (ESR) and serum C-reactive protein (CRP) testing in all the patients (7). Radiographs are also routinely obtained in the suspected PJI work-up. Whether joint aspiration is required is then decided based on ESR/CRP results and PJI probability (7). AAOS guidelines also state that positron emission tomography/computed tomography (PET/CT) could be used in certain patients, and over the past decade, several studies have assessed the utility of PET/CT for diagnosing PJI (8–10). The results of previous studies have varied from report to report. FDG PET/CT use may reduce diagnostic procedure durations, thereby improving the quality of care. Moreover, the early diagnosis of PJI can lead to more effective therapeutic management. No up-to-date meta-analysis that assesses the diagnostic accuracy of FDG PET/CT currently exists, with the most recent one having been published in 2010 (11). The present study therefore aimed to perform a pooled analysis on all available literature concerning the diagnostic accuracy of FDG PET/CT for PJI.

## Material and Methods

### Eligibility Criteria

#### Included Study Types

This study included all studies examining the diagnostic accuracy of FDG PET/CT for PJI regardless of study design, participant characteristics, and assessed PJI type. We only included studies that reported the sensitivity and specificity of employed diagnostic techniques or provided sufficient data to calculate those values. Studies without accessible full-text manuscripts were excluded. Case reports and studies with sample sizes under 10 were also excluded.

#### Index Test

This study included studies examining the diagnostic accuracy of FDG PET/CT for PJI.

#### Reference Standards

We included studies only if the diagnostic accuracy of FDG PET/CT was compared with that of an intraoperative positive culture, regardless of whether it was combined with histopathological evidence concerning periprosthetic tissue acute inflammation caused by surgical debridement or prosthesis removal and/or the presence of the sinus tract that communicates with the prosthesis.

#### Outcome Measures

The number of patients who were true positives, false positives, true negatives, and false negatives for PJI.

### Search Strategy

We conducted a comprehensive, systematic, and extensive search of electronic databases including PubMed Central, EMBASE, MEDLINE, SCOPUS, and the Cochrane Library. We used both medical subject headings (MeSH) and free-text words to query all searched databases. Keywords and their synonyms were employed using appropriate truncations, wildcards, and proximity searching. The following MeSH terms and free text terms were used in various combinations: “Validation Studies”, “Periprosthetic Joint Infection”, “Positron Emission Tomography/Computed Tomography”, “PET/CT”, “^18^FDG PET/CT”, “Fluoride PET/CT”, “Histopathology” “Sensitivity”, “Specificity”, “Diagnosis”, and “Diagnostic Accuracy Studies”. Searches were also conducted in each database for key concepts using corresponding subject headings. The final search was carried out by combining individual search results using the appropriate Boolean operators (“OR” and “AND”). Only publications published prior to February 2021 and published in the English language were considered.

### Study Screening

**Figure 1:**
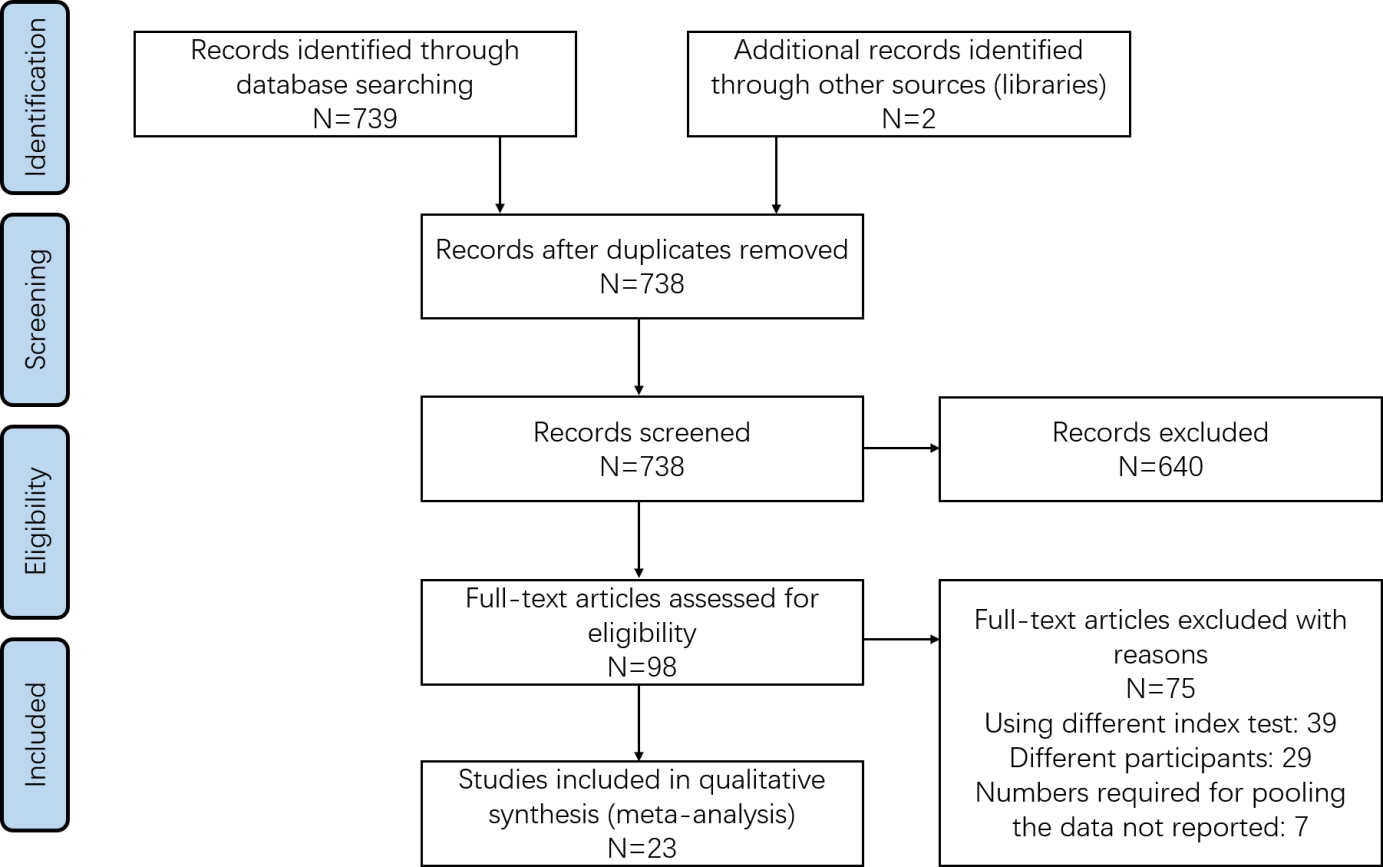
Search strategy. Flowchart was made as per PRISMA guidelines.

Preliminary screening, involving title and abstract assessment, was performed by two reviewers. Here, all hits returned by search queries were imported to a specified Endnote library. After duplicates were removed, the library was manually scanned to identify short-list candidates. The full-text articles were retrieved for these shortlisted studies and reviewed by the same two reviewers. Shortlisted studies not satisfying eligibility criteria were excluded, with the reason for exclusion noted. Any disagreements between the two reviewers were resolved through arbitration with a third investigator. This process is outlined in Figure 1 and took place in accordance with PRISMA guidelines (12).

### Data Extraction and Management

Study data was extracted using a pre-defined data extraction form. Extracted data included design, setting, index test, reference standards (gold standard), PJI site, sample size, average age, inclusion and exclusion criteria, test positives (true & false), and test negatives (true & false). Data was transferred into STATA software. Data quality was checked and verified by the third investigator who arbitrated disputes during the study screening process

### Bias Risk Assessment

Two independent reviewers assessed bias risk in included studies using the “Quality Assessment of Diagnostic Accuracy Studies-2 (QUADAS-2) tool” (13). The following domains were examined: patient selection, index tests, reference standards, flow and timing of assessments. Grades were assigned as high, low, and unclear for each domain.

### Statistical analysis

The meta-analysis was performed using STATA software version 14.2 (StataCorp, College Station, TX, USA) to obtain pooled sensitivity, specificity, positive likelihood ratio (LRP), negative likelihood ratio (LRN), and summary diagnostic odds ratio (DOR) values for PET/CT. Summary Receiver Operator Characteristic curves (sROC) were constructed and summarized as area under the curve (AUC). Study-specific and pooled estimates were graphically represented through Forest plots. A Fagan plot was constructed to demonstrate how much a PET/CT result changes the probability that a patient has PJI. LR scattergram was used to determine the clinical value of FDG PET/CT. The presence of between-study variance due to heterogeneity was assessed using three methods: graphical representation via a bivariate box plot, the chi square test for heterogeneity, and *I*^2^ statistics to quantify inconsistency (<25%: mild, 25%–75%: moderate, >75%: substantial). Subgroup analysis and meta-regression was performed as well. Publication bias was assessed and graphically represented using a funnel plot, with the asymmetry of the plot tested using Deek’s test.

## Results

### Study Selection

The literature search revealed 739 unique articles, and 98 were shortlisted for full-text retrieval. We also retrieved full-texts for two additional articles found by screening references cited by other retrieved studies. A total of 23 studies, containing information on 1,437 patients, met inclusion criteria and were used for meta-analysis (Figure 1) (8–10, 14–33).

### Characteristics of Included Studies

Twenty out of 23 studies were prospective. The USA was the most represented setting, with six studies conducted in that country. They were followed by Germany (5) and India (2). The mean age of study participants within individual studies ranged from 53.0 to 76.4 years. Sample sizes in individual studies ranged from 17 to 221 patients. Fourteen studies assessed patients with suspected PJI in the hip, while seven looked at PJI in both the hip and knee. Most of the studies used a combination of intraoperative findings with histopathological, microbiological, and clinical examinations as the reference standard (Table 1).

**Table 1:** Characteristics of the included studies (*N*** **=** **23).

Study No	First author and year	Country	Study design	Sample size	Study participants	Site of PJI	Type of PET/CT	Reference standard	Mean age (in years)
1	Aksoy et al. 2013 (14)	Turkey	Prospective	54	Patients with prostheses (knee & hip prostheses) who were suspected for PJI	Hip & Knee	FDG PET/CT	Postoperative histopathological/microbiological examination or clinical work-up	61
2	Basu et al. 2014 (8)	USA	Prospective	221	Patients with painful hip or knee arthroplasty, who were scheduled to undergo clinical and diagnostic evaluation for prosthesis revision	Hip & Knee	FDG PET/CT	Diagnosis confirmed upon either detection of microorganisms in cultures or purulent fluid within area of interest and presence of neutrophilic infiltrates at sites	57
3	Chacko et al. 2002 (15)	USA	Prospective	41	Patients with hip arthroplasty suspected for PJI	Hip	FDG PET/CT	Final diagnosis based on microbiology, histopathology, surgical & clinical follow-up	61.9
4	Chen et al. 2010 (16)	Taiwan	Prospective	24	Patients with painful hip prosthesis or those with an interim hip spacer following resection arthroplasty	Hip	FDG PET/CT	Intraoperative tissue cultures, intraoperative pathology, and clinical follow-up	Not reported
5	Chryssikos et al. 2008 (17)	USA	Prospective	127	Patients with painful hip prosthesis	Hip	FDG PET/CT	Combination of preoperative tests, intraoperative findings, histopathology, clinical followup	59
6	Delank et al. 2006 (18)	Germany	Prospective	36	Patient scheduled for revision surgery for hip or knee prosthesis	Hip & Knee	FDG PET/CT	Intraoperative findings, histopathology, microbiological investigations	Not reported
7	Falstie-Jensen et al. 2019 (19)	Denmark	Prospective	86	Patients with failed shoulder arthroplasty	Shoulder	FDG PET/CT	Positive cultures in at least three of five specimens	67
8	Garcia-Barrecheguren et al. 2007 (20)	Spain	Prospective	24	Patients with hip replacement prosthesis	Hip	FDG PET/CT	Intraoperative findings, histopathology, microbiological investigations	67.8
9	Kiran et al. 2019 (21)	UK	Prospective	130	Patients with painful unilateral cemented total hip arthroplasty	Hip	FDG PET/CT	Histopathology / microbiological culture	67.5
10	Kumar et al. 2016a (22)	India	Prospective	45	Patients with painful hip prosthesis	Hip	F-PET/CT	Intraoperative findings, histopathology, microbiological investigations	54
11	Kumar et al. 2016b (23)	India	Prospective	42	Patients with painful hip prosthesis	Hip	F & FDG PET/CT	Intraoperative findings, histopathology, microbiological investigations	53
12	Kwee et al. 2017 (9)	Netherlands	Retrospective	78	Patients with painful hip prosthesis	Hip	FDG PET/CT	Culture results at revision surgery	66.5
13	Love et al. 2004 (24)	USA	Retrospective	59	Patients with painful, failed lower extremity joint prosthesis	Hip & Knee	FDG PET/CT	Intraoperative findings, histopathology, microbiological investigations	Not reported
14	Manthey et al. 2002 (25)	Germany	Prospective	23	Patients with painful hip or knee prosthesis	Hip & Knee	FDG PET/CT	Positive culture results following surgery	70
15	Mayer-Wagner et al. 2010 (26)	Germany	Prospective	49	Patients with lower limb arthroplasty complaints	Hip & Knee	FDG PET/CT	Positive microbiological culture results following surgery	Not reported
16	Mumme et al. 2005 (27)	Germany	Prospective	70	Patients with hip arthroplasty	Hip	FDG PET/CT	Intraoperative findings, histopathology, microbiological investigations	68.7
17	Pill et al. 2006 (28)	USA	Prospective	92	Patients with painful hip prosthesis	Hip	FDG PET/CT	Clinical examination and preoperative and intraoperative findings	Not reported
18	Reinartz et al. 2005 (29)	Germany	Prospective	92	Patients with painful hip arthroplasty	Hip	F-PET/CT	Laboratory test, radiological examination and clinical examination	68
19	Stumpe et al. 2004 (30)	Switzerland	Prospective	35	Patients with painful hip arthroplasty	Hip	FDG PET/CT	Microbiological evaluation of surgical specimens	64
20	Van Acker et al. 2001 (31)	Belgium	Prospective	21	Patients with painful total knee arthroplasty	Knee	FDG PET/CT	Operative findings, culture and clinical outcome	66
21	Vanquickenborne et al. 2003 (32)	Belgium	Prospective	17	Patients with painful hip prosthesis	Hip	FDG PET/CT	Bacteriology of samples obtained by surgery or by needle aspiration and/or clinical findings	62
22	Verberne et al. 2018 (10)	Netherlands	Retrospective	33	Patients with painful hip prosthesis	Hip	FDG PET/CT	Pre-operative and intra-operative findings with clinical follow-up > 12 months	76.4
23	Zhuang et al. 2001 (33)	USA	Prospective	38	Patients in whom infection was suspected after artificial hip or knee placement	Hip & Knee	FDG PET/CT	Surgical exploration or clinical follow-up for 1 year	Not reported

*USA, United States of America; UK, United Kingdom; F- PET/CT, 18Fluoride Positron Emission Tomography/Computed Tomography; FDG PET CT, 18Fluorodeoxyglucose Positron Emission Tomography/Computed Tomography; PJI, Prosthetic Joint Infection*.

### Risk of Bias Assessment

**Figure 2:**
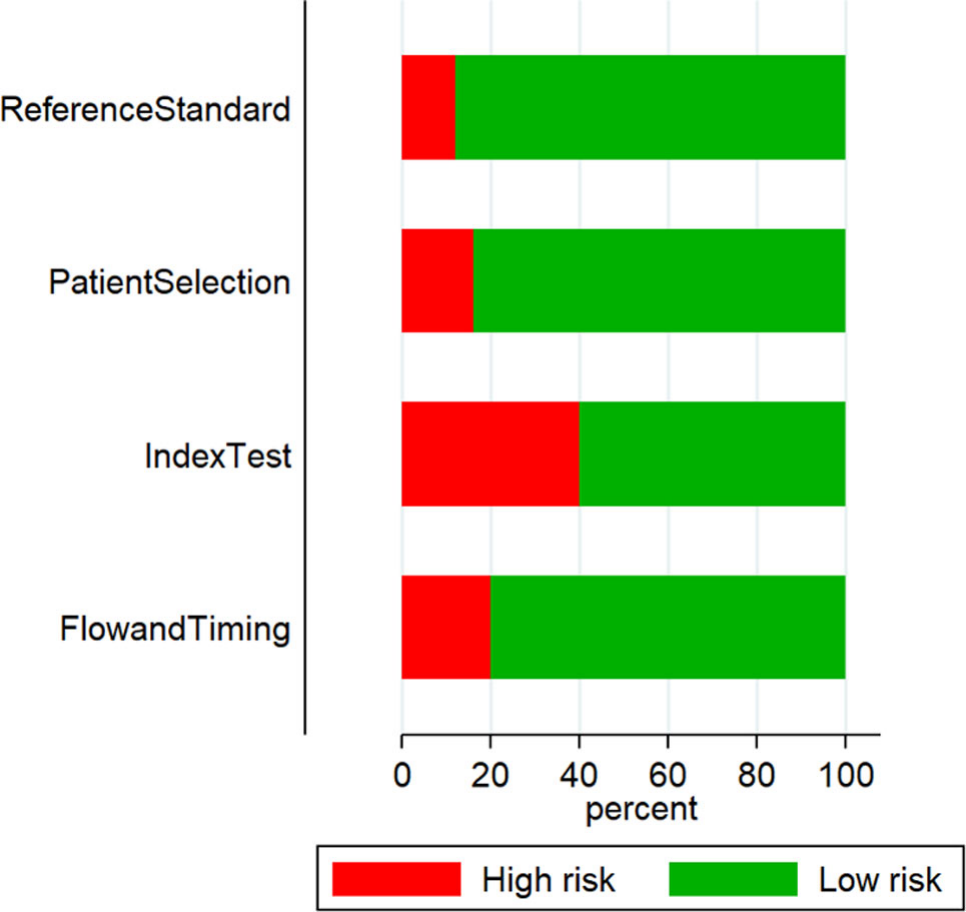
Study quality assessment using the QUADAS-2 tool (*n* = 23).

QUADAS tool results found that 3 out of 23 studies had a high risk of patient selection bias, 10 had a high risk of conduct and interpretation of index test bias, 5 had a high risk of patient flow and interval between index tests and reference standards bias, and 2 had a high risk of reference standard bias (Figure 2 and Table 2).

**Table 2:** Quality assessment of the included studies (*N* = 23).

Study No	First author and year	Patient selection	Index test	Reference standard	Flow and timing
1	Aksoy et al. 2013 (14)	Low Risk	Low Risk	Low Risk	Low Risk
2	Basu et al. 2014 (8)	Low Risk	High Risk	Low Risk	Low Risk
3	Chacko et al. 2002 (15)	Low Risk	Low Risk	Low Risk	Low Risk
4	Chen et al. 2010 (16)	Low Risk	High Risk	Low Risk	High Risk
5	Chryssikos et al. 2008 (17)	Low Risk	High Risk	Low Risk	Low Risk
6	Delank et al. 2006 (18)	Low Risk	Low Risk	Low Risk	Low Risk
7	Falstie-Jensen et al. 2019 (19)	Low Risk	Low Risk	Low Risk	Low Risk
8	Garcia-Barrecheguren et al. 2007 (20)	Low Risk	High Risk	Low Risk	Low Risk
9	Kiran et al. 2019 (21)	Low Risk	High Risk	Low Risk	Low Risk
10	Kumar et al. 2016a (22)	Low Risk	Low Risk	Low Risk	Low Risk
11	Kumar et al. 2016b (23)	Low Risk	Low Risk	Low Risk	Low Risk
12	Kwee et al. 2017 (9)	High Risk	High Risk	High Risk	High Risk
13	Love et al. 2004 (24)	High Risk	High Risk	Low Risk	High Risk
14	Manthey et al. 2002 (25)	Low Risk	Low Risk	Low Risk	Low Risk
15	Mayer-Wagner et al. 2010 (26)	Low Risk	Low Risk	Low Risk	Low Risk
16	Mumme et al. 2005 (27)	Low Risk	High Risk	High Risk	High Risk
17	Pill et al. 2006 (28)	Low Risk	Low Risk	Low Risk	Low Risk
18	Reinartz et al. 2005 (29)	Low Risk	High Risk	Low Risk	Low Risk
19	Stumpe et al. 2004 (30)	Low Risk	Low Risk	Low Risk	Low Risk
20	Van Acker et al. 2001 (31)	Low Risk	Low Risk	Low Risk	Low Risk
21	Vanquickenborne et al. 2003 (32)	Low Risk	Low Risk	Low Risk	Low Risk
22	Verberne et al. 2018 (10)	High Risk	High Risk	Low Risk	High Risk
23	Zhuang et al. 2001 (33)	Low Risk	Low Risk	Low Risk	Low Risk

### Diagnostic Accuracy of FDG PET/CT for PJI

**Figure 3:**
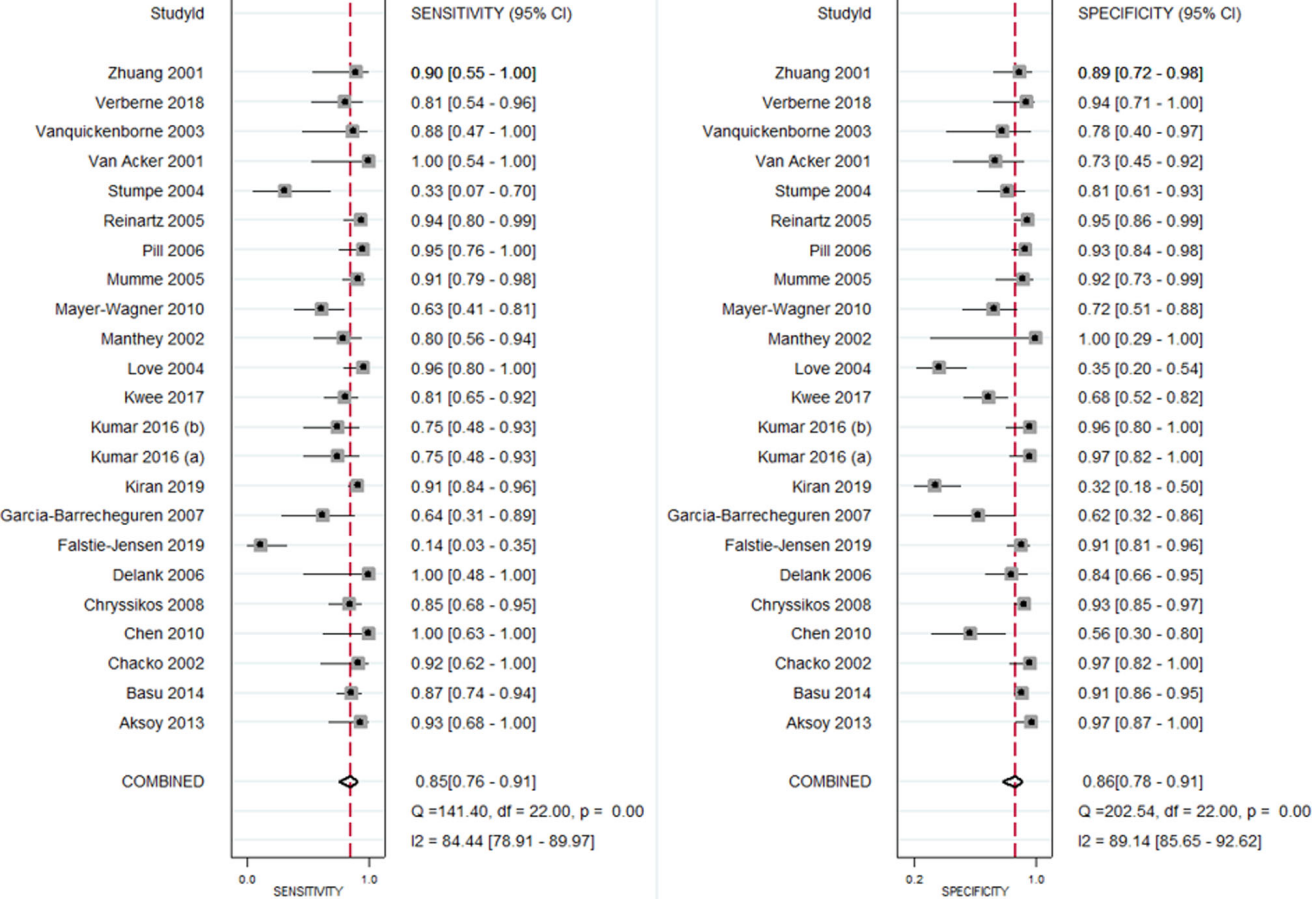
Forest plot showing pooled sensitivity and specificity for PET/CT.

**Figure 4:**
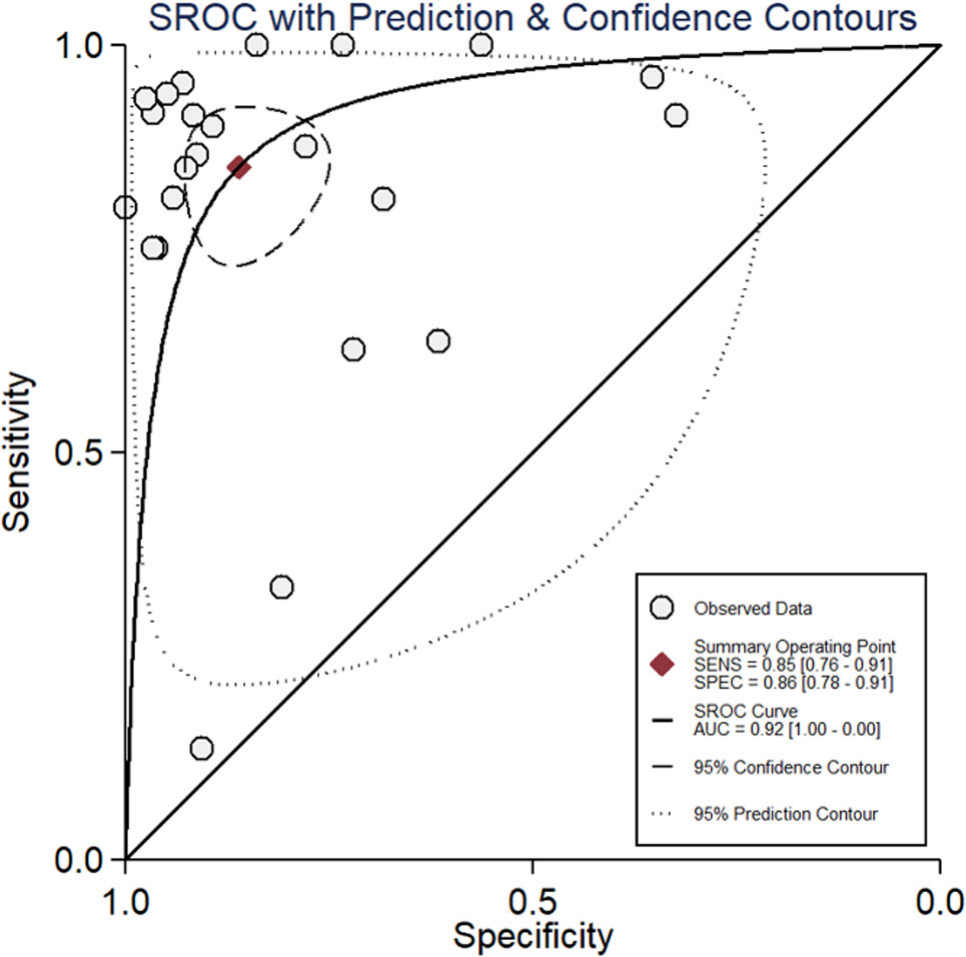
SROC Curve for PET/CT for diagnosing PJI.

**Figure 5:**
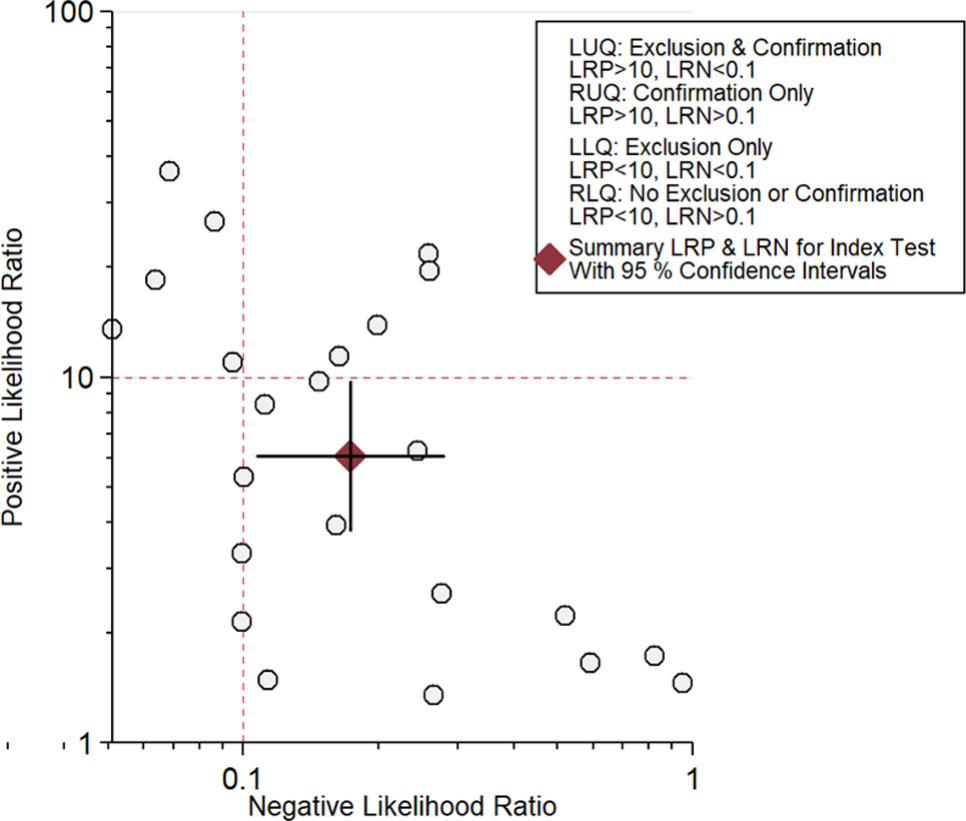
Likelihood scattergram for PET/CT.

**Figure 6:**
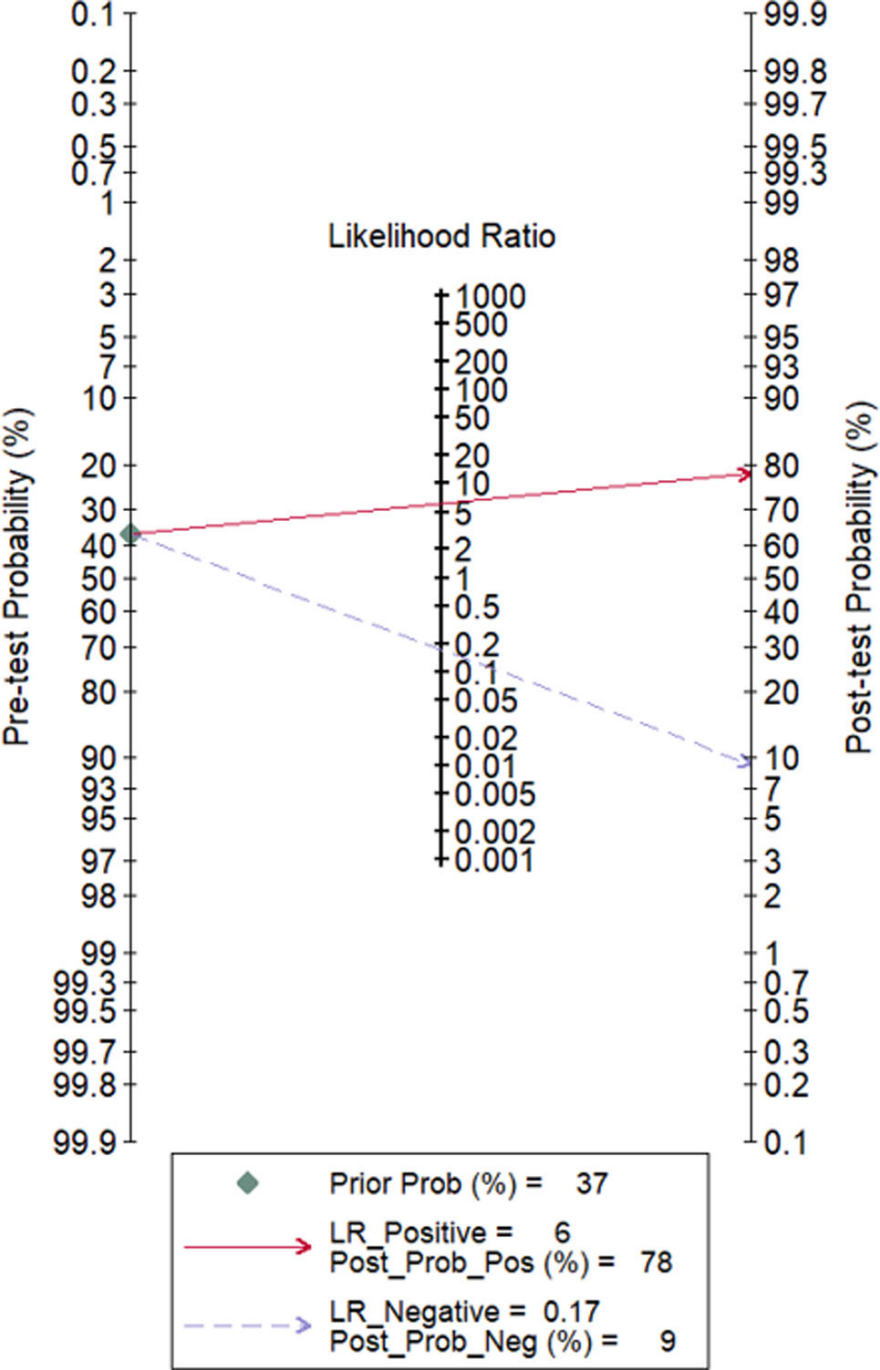
Fagan nomogram evaluating the overall value of PET/CT for PJI diagnosis.

**Figure 7:**
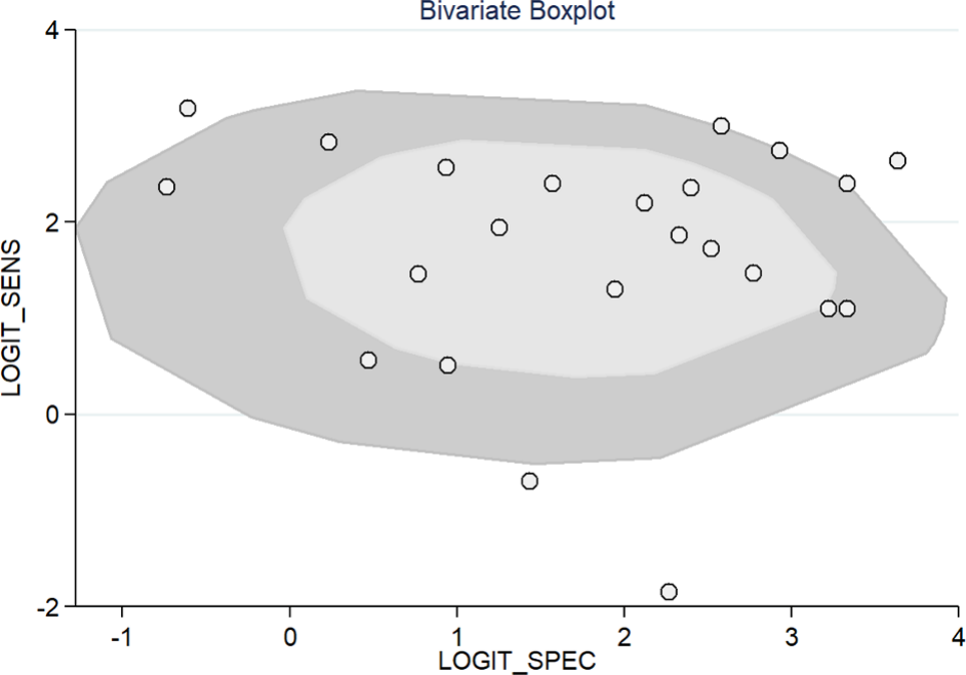
Bivariate boxplot showing sensitivity and specificity for included studies.

All 23 included studies reported on the utility of FDG PET/CT for diagnosing PJI (8–10, 14–33). Pooled sensitivity and specificity of FDG PET/CT for diagnosing PJI were 85% (95% CI, 76%, 91%) and 86% (95% CI, 78%, 91%), respectively, with an AUC value of 0.92 (Figures 3, 4). The DOR was 35 (95% CI, 17, 74), LRP was 6.1 (95% CI, 3.8, 9.7), and LRN was 0.17 (95% CI, 0.11, 0.28). LR scattergram (Figure 5) showed LRP and LRN in the right lower quadrant, indicating that the PET/CT cannot be used for confirmation or exclusion. Figure 6 shows a high clinical utility of PET/CT for diagnosing PJI (Positive: 78%; Negative: 9%), differing significantly from the pre-test probability (37%). We also found significant inter-study variability (heterogeneity) with a chi-square *p* value <0.001 and an *I*^2 ^> 75%. The bivariate box plot further confirmed this heterogeneity (Figure 7).

**Figure 8:**
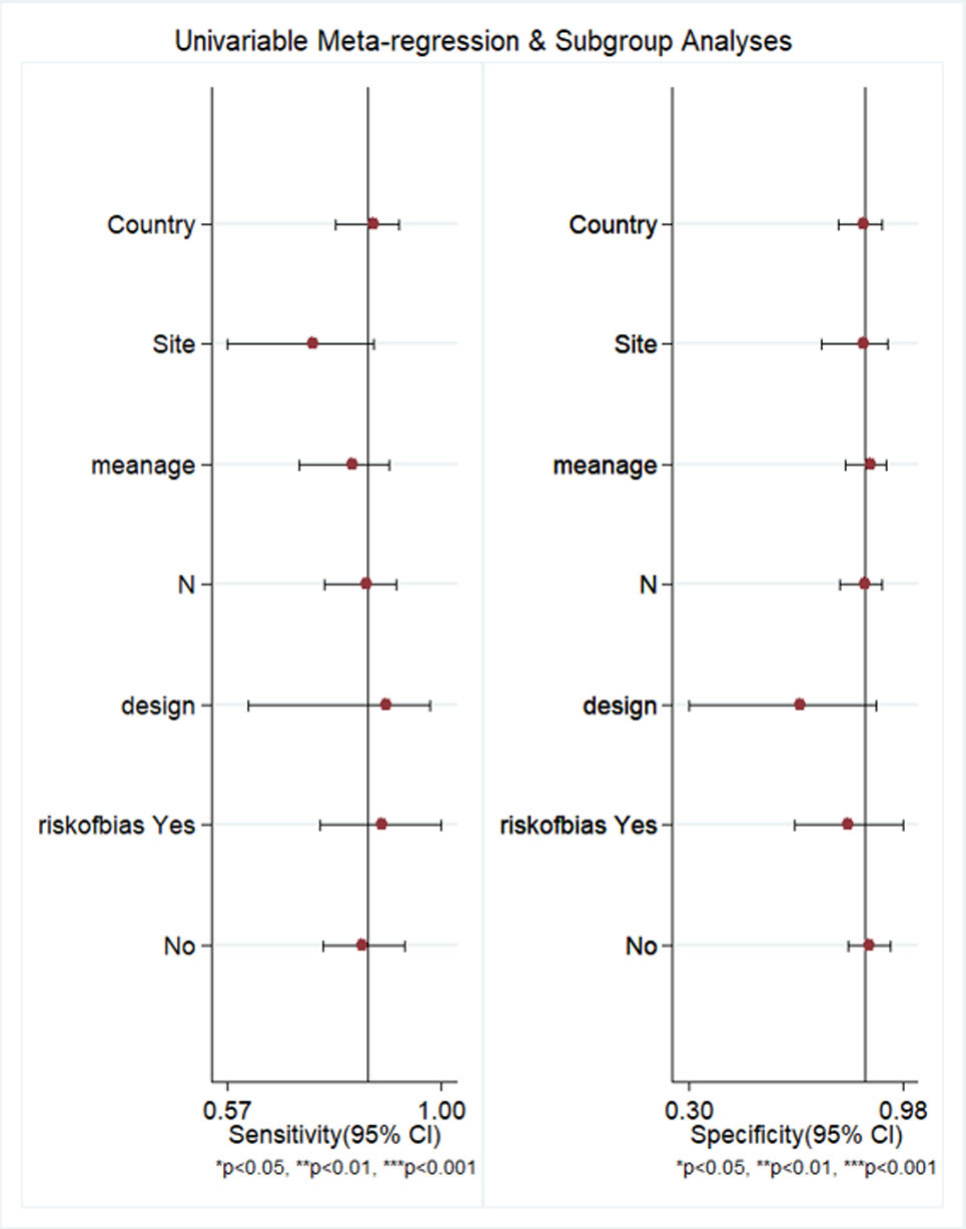
Meta-regression for ascertaining sources of heterogeneity.

**Figure 9:**
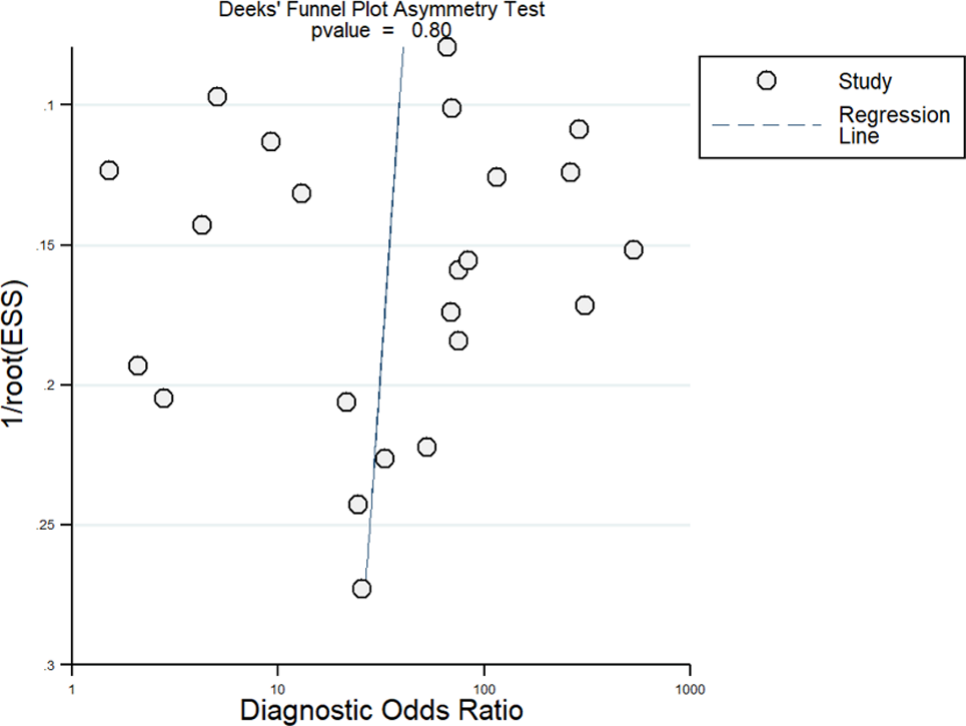
Funnel plot for publication bias.

We performed meta-regression to find heterogeneity sources, using factors such as study design, PJI site, country, sample size, mean age, and quality related factors (Figure 8). However, we could not find any factors to be significantly associated using the sensitivity and specificity model, while only mean age (*p* < 0.001) was found to be a source of heterogeneity using the joint model. Deek’s test showed a non-significant *p*-value (*p* = 0.80), thus indicating the absence of publication bias. This was confirmed by the symmetrically-shaped funnel plot (Figure 9).

Subgroup analysis delineating based on study design type revealed that prospective studies alone had similar pooled sensitivity (85%) and specificity (88%) values relative to the entire dataset. Studies possessing low bias risk had higher specificity (87%) compared to studies with high bias risk (81%). However, we did not find any significant difference in specificity between low-bias risk and high-bias risk studies (85% in both subgroups). Subgroup analysis delineating based on PJI site found that the hip location possessed similar sensitivity (87%) and specificity (85%) relative to the overall estimate. Insufficient sample size prevented subgroup analysis on other locations.

## Discussion

The diagnostic approach for patients with suspected PJI has varied considerably across different healthcare centers globally and depends on the experience of the health professional and the availability of the latest technological equipment (34). Presently, radiography is widely used as an initial diagnostic protocol, with PET/CT a popular modality for its reported diagnostic accuracy (25–33). However, this accuracy has not been confirmed through a systematic evaluation. Hence, our goal here was to determine the diagnostic accuracy of FDG PET/CT for PJI.

Our systematic literature search found 23 studies reporting the utility of FDG PET/CT for diagnosing PJI. We found a high pooled sensitivity (85%) and specificity (86%) for FDG PET/CT in PJI diagnosis. Moreover, the clinical utility of FDG PET/CT was demonstrated by how Fagan’s nomogram showed a significant rise in post-PET/CT probability compared to pre-PET/CT probability. Our findings are similar to those previously reported (11, 35–37). Over the past few years, PET/CT has been used as a standard scan system for PET in several medical centers around the world. A metallic prosthesis following the surgery can produce a strong artifact in the CT images, resulting in underestimation or overestimation of the concentration of activity around these metallic prostheses. Hence, correction of PET images by CT-based attenuation provides accurate images and misinterpretation of the image tracer accumulation can be reduced using the newer metal artifact reduction systems (8, 38).

We also noted that FDG PET/CT diagnostic efficacy did not differ significantly depending on study bias and PJI site. Certain studies have reported that labeled leukocyte/marrow imaging is a superior diagnostic tool to FDG PET/CT (24). The possible reason for this finding is that the indication for PET-CT in PJI is limited to special situations in which a painful implant may stem from aseptic or septic loosening. If joint aspiration is dry or the surgeon wants to gather information on the whole implant, imaging modalities gain importance. In those revision situations which usually involve older patients, time of imaging and radiation exposure are not of utmost interest. Thus, WBC scintigraphy is the more accurate option, if available. In addition, FDG PET/CT is not a part of standard definition of protocol for PJI diagnosis. This is mainly because it is considered that there is no place of nuclear imaging for acute infections. However, FDG PET/CT has certain advantages in terms of feasibility, availability, and logistics (requiring only one radiotracer injection). Thus, PET/CT can be added as part of the standard diagnostic protocol for PJI if WBC scintigraphy is unavailable and diagnosis needs imaging.

Our review has certain strengths. This meta-analysis involved a large number of studies with high sample sizes (23 studies with >1,400 participants). Most included studies had high quality across most of the domains under the QUADAS-2 tool, and we did not find any significant publication bias, which further adds to the credibility of results in our analysis. Deek’s test results and funnel plot showed a possibility of the absence of a significant publication bias. However, there are several limitations to this meta-analysis. First, we found significant inter-study heterogeneity, limiting our ability to interpret or infer the pooled results. Although we investigated potential reasons for such high heterogeneity using meta-regression, we could not identify any factors other than mean age. Second, FDG PET/CT diagnostic accuracy in practice depends on various factors, such as assessment timing, PJI site, number and experience of interpreters, FDG dose, time interval between FDG administration and scanning and additional patient co-morbidities. However, we could not assess the influence of any of these factors due to a lack of available data.

Despite these shortcomings, our findings provide valuable information and important implications for the clinical management of PJI and suggest that FDG PET/CT can be used as an effective screening and diagnostic tool. Moreover, early diagnoses of PJI can further lead to the more effective therapeutic management of the diagnosed patients. Further updated reviews should compare the diagnostic performance of PET/CT with other similar imaging techniques. In addition, large-scale longitudinal studies are required to check the diagnostic accuracy of PET/CT based on the different sites of PJI because most available studies have hip as the site of PJI and limited study available on knee and shoulder PJI.

## Conclusion

Our study found that can have an important role as a diagnostic tool in certain situations of PJI, given its high sensitivity and specificity. However, the finding should be interpreted with caution given the higher level of heterogeneity. In the future, studies should seek to compare the diagnostic performance of FDG PET/CT with other similar imaging techniques. Similarly, large-scale longitudinal studies are required to examine the diagnostic accuracy of FDG PET/CT for different PJI sites.

## Data Availability

The original contributions presented in the study are included in the article/Supplementary Material, further inquiries can be directed to the corresponding author/s.
